# Comparison of stigmatizing views towards COVID-19 and mental disorders among adolescent and young adult students in China

**DOI:** 10.3389/fpsyt.2023.1170540

**Published:** 2023-07-06

**Authors:** Tian-Ming Zhang, Xin-Feng Zhang, Xian-Dong Meng, Yi Huang, Wei Zhang, Hui-Hui Gong, Sherry Kit Wa Chan, Xiao-Chuan Chen, Ru Gao, Roberto Lewis-Fernández, Yuan-Yuan Fan, Chang-Cheng Liu, Lu Huang, Xiao-Peng Deng, Bo Liu, Mao-Sheng Ran

**Affiliations:** ^1^Department of Social Work, Shanghai University, Shanghai, China; ^2^Jingzhou Mental Health Center and The Mental Health Institute of Yangtze University, Jingzhou, Hubei, China; ^3^Mental Health Center, West China Hospital, Sichuan University, Chengdu, Sichuan, China; ^4^School of Public Administration, Hunan University, Changsha, Hunan, China; ^5^Department of Psychiatry, School of Clinical Medicine, LKS Faculty of Medicine, The State Key Laboratory of Brain and Cognitive Sciences, The University of Hong Kong, Hong Kong, Hong Kong SAR, China; ^6^Ya'an Fourth People's Hospital, Ya'an, Sichuan, China; ^7^Wenjiang People‘s Hospital, Chengdu, Sichuan, China; ^8^Columbia University and New York State Psychiatric Institute, New York, NY, United States

**Keywords:** stigma, COVID-19, mental illness, students, China

## Abstract

**Objective:**

Infectious diseases including COVID-19 and mental disorders are two of the most common health conditions associated with stigma. However, the comparative stigma of these two conditions has received less attention in research. This study aimed to compare the prevalence of stigmatizing views toward people with COVID-19 and mental disorders and the factors associated with these views, among a large sample of adolescent and young adult students in China.

**Methods:**

A total of 9,749 adolescents and young adults aged 15–24 years completed a survey on stigmatizing attitudes toward COVID-19 and mental disorders, as well as mental health-related factors, including general mental health status and symptoms of depression, anxiety, insomnia, and post-traumatic stress disorder (PTSD). Multivariable linear regression analyses were conducted to identify factors associated with stigmatizing views.

**Findings:**

The prevalence of COVID-19 and mental disorders-related stigma was 17.2% and 40.7%, respectively. COVID-19-related stigma scores were significantly higher among male students (β = 0.025, *p* < 0.05), those without quarantine experience (β = 0.035, *p* < 0.001), those with lower educational level (*p* < 0.001), those with lower family income (*p* < 0.01), and those with higher PTSD symptoms (β = 0.045, *p* < 0.05). Mental disorder-related stigma scores were significantly higher among individuals with average and lower-than-average levels of family income (*p* < 0.01), depression symptoms (β = 0.056, *p* < 0.001), anxiety symptoms (β = 0.051, *p* < 0.001), and mental health problems (β = 0.027, *p* < 0.05).

**Conclusion:**

The stigma of mental disorders is higher in the youth population than the stigma of COVID-19. Factors associated with stigmatizing attitudes toward people with COVID-19 and mental disorders varied across the youth. Stigma-reduction interventions among the youth should be targeted specifically to COVID-19 or mental disorders conditions.

## Introduction

Health-related stigma has become a major public health issue globally due to its detrimental consequences, such as preventing people from seeking professional treatment, and is considered a hidden disease burden (1). People with health conditions such as infectious diseases and chronic mental disorders often face societal stigmatization resulting from strong negative feelings and beliefs about those conditions (2). Health-related stigma has been conceptualized as a social process of experiencing rejection, isolation, discrimination, or devaluation because of unreasonable social feelings about individuals with a specific health condition (3).

As with other newly emerging infectious diseases, the unprecedented COVID-19 outbreak has also evoked fear, psychological distress, and social discrimination toward the infected individuals in addition to tremendous physical health consequences (4, 5). Given its elevated transmissibility and morbidity, COVID-19-related stigmatization is pervasive and poses a serious threat to individuals associated with the disease, such as healthcare providers, infected people, and survivors. Moreover, Asians living in Western countries are also at risk of stigmatization because the illness was first identified in China (6, 7).

Previous studies have documented the prevalence of having stigmatizing ideas about COVID-19 in community settings ranging from 5.2 to 73.3% (e.g., healthcare workers) (8). Evidence suggested that people infected with COVID-19 not only feel ostracized but also internalize public stigma and thus are reluctant to reconnect with society after the end of their quarantine period (9). Given the ramifications of COVID-19-related stigma, a growing number of studies have emerged, assessing its psychosocial correlates, including health literacy about COVID-19, media broadcasts of biased information about COVID-19, fear and anxiety about contracting COVID-19, and quarantine and social-distancing policies (10–12). Moreover, several demographic factors, including gender, age, education level, and employment status, have been associated with COVID-19-related stigma (13).

A prolonged COVID-19 pandemic is also likely to elevate the risk of mental disorders (14). Given containment strategies such as lockdowns, social distancing policies, and quarantines, the levels of anxiety, depression, and sleep quality in the general population have worsened during the pandemic (15). The prevalence of mental disorders has increased by 10% in the Czech Republic and by 20% in India since the COVID-19 outbreak (16). Among the different stigmas toward health-related conditions, mental disorder-related stigma is very common and most widely investigated (17). Numerous studies have shown that people with mental disorders are observed as dangerous, irresponsible, and childlike (18); are often shunned by friends, relatives, and community residents (19, 20); and face enacted discrimination in the workplace (21).

Although both COVID-19 and mental disorders are two of the most common health conditions associated with stigma in the general population, inconsistent attitudes are linked to the two diseases because of their specific characteristics and those of individuals afflicted by them. Previous studies have compared the public stigma associated with diverse infectious diseases, such as HIV, SARS, tuberculosis, and Ebola, indicating that HIV-related stigma was the highest (22), or have compared the stigma associated with two different mental disorders (23). Another previous study has indicated that stigmatizing attitudes are related to the features of diseases, for instance, the threat of diseases to others and restrictions imposed by the diseases on the body (24). Given the divergent attributes of COVID-19 and mental disorders, the public may hold various attitudes toward those two diseases. It will be crucial to understand the differences between the two types of stigmas for improving further anti-stigma interventions. However, there are scarce studies that have compared the stigmatizing views toward COVID-19 and mental disorders. Although children and adolescents have experienced severe mental health problems during the COVID-19 pandemic (25), few studies have focused on COVID-19-related stigmatization in this vulnerable group. The youth rarely use mental health services, even when they have psychiatric problems, and may hold more negative attitudes toward people with mental disorders than older adults (26). A review study has shown various detrimental mental health stigma-related psychosocial outcomes (e.g., poorer mental health status, social rejection, and concealment of substance abuse) among young people (27). Thus, in this study, we compared stigmatizing views toward people with COVID-19 and mental disorders among a large sample of community adolescent students in China. This study aimed to compare the prevalence of stigmatizing views toward people with COVID-19 and mental disorders and also the factors associated with these views among a large sample of adolescent and young adult students in China.

## Methods

### Study design, participants, and procedures

Data were collected from a convenience sample of adolescent and young adult students from 1 June 2021 to 6 January 2022 in Jingzhou City, located in Hubei province, not far from Wuhan, the outbreak epicenter of COVID-19 in China. During the period of data collection, there were only two confirmed COVID-19 cases in Jingzhou City. A self-report questionnaire was designed to investigate the mental health status of adolescent students during the COVID-19 pandemic and delivered through an online platform (https://www.wjx.cn/), a widely used online survey tool in China. The questionnaire was also available in print. We distributed the questionnaire to nine educational institutions, including two universities, three junior colleges, and four middle and high schools. The informed consent form explained the purpose and significance of the study and was displayed on the first page of the questionnaire. For participants under the age of 18 years, besides participants' informed consent, we also consulted their parents and/or teachers for verbal informed consent before they filled out questionnaires. A total of 14,638 students completed the questionnaire. After excluding those who were not within the age range of 15–24 years, the final sample included 9,749 participants. Ethical approval was obtained from the medical ethics committee of the Jingzhou Mental Health Center.

### Measures

Self-reported sociodemographic characteristics of participants included gender (male and female), age group (15–17 and 18–24 years), educational attainment (junior high school, senior high school, college, and undergraduate), family income (below average, average local household income, and above average), one-child status, quarantine condition, and self-report history of mental disorders.

Stigma toward COVID-19-infected individuals was assessed with an 11-item scale developed by our research team. Six scale items were generated from existing measures of public stigma toward mental disorders (28), and five items were based on an existing measurement of stigma toward recovered SARS patients (29). Each item was rated on a 4-point Likert scale (1 = strongly disagree, 2 = disagree, 3 = agree, and 4 = strongly agree), with higher scores indicating a higher level of stigma (range: 11–44). Sample items include “I am worried that people with COVID-19 will cause harm to others” and “People with COVID-19 should stay away from others, even if he/she has been cured.” The internal consistency of the measure was very good in this sample (Cronbach's α = 0.878).

Stigma toward people with mental disorders was measured with a modified version of Link's Perceived Discrimination-Devaluation Scale [LPDDS; (30)]; its 13 items were scored using a 4-point Likert scale (1 = strongly agree, 2 = agree, 3 = disagree, and 4 = strongly disagree), with higher scores indicating higher stigmatization (range: 13–52). The Chinese version of this instrument has acceptable reliability among the general population (18). Its internal consistency was α = 0.741 in our sample.

Mental health-related factors assessed in this study consisted of general mental health status and symptoms of depression, anxiety, insomnia, and post-traumatic stress disorder (PTSD), assessed as follows:

a) The 12-item General Health Questionnaire (GHQ-12) was used to measure participants' general mental health status over the recent few weeks (31). Each item in the GHQ-12 was rated on a 4-point Likert scale ranging from 0 to 3. Responses were summed to form a total score (range: 0–36), with higher scores indicating more severe mental health problems. A cutoff score of 3 indicates potential mental health problems. The Chinese version of GHQ-12 has been widely used in general population samples including adolescents (32). Internal consistency was α = 0.843 for the GHQ-12 in this sample.
b) The 9-item Patient Health Questionnaire (PHQ-9) and the 7-item Generalized Anxiety Disorder (GAD-7) scale were used to assess participants' depression and anxiety status over the past 2 weeks (33). Each item in the PHQ-9 and GAD-7 was rated on a 4-point Likert scale (0 = not at all;1 = several days; 2 = more than half the days; and 3 = nearly every day). Scores were summed for each scale (ranges: PHQ-9 = 0–27; GAD-7 = 0–21), with a higher score indicating greater severity of depression or anxiety. Scores of more than 5 were considered potentially clinically relevant for depression or anxiety. Both scales have been validated among Chinese populations and adolescents (34, 35). The Cronbach's α for PHQ-9 and GAD-7 in the present study were 0.920 and 0.940.
c) The 7-item Insomnia Severity Index (ISI-7) was used to measure participants' type and symptoms of sleep disturbances over the past 2 weeks (36). A 5-point Likert scale was used to rate the scale (range: 0–4), with higher scores indicating greater insomnia. A cutoff of 8 identified clinically relevant insomnia for the current study. The ISI-7 has been widely used in epidemiological research (37). A Chinese version of the scale was validated among Chinese adolescents and young adult students in earlier studies (35). Internal consistency was α = 0.899 for the ISI-7 in this sample.
d) The Post-traumatic Checklist for DSM-5 (PCL-5) assessed DSM-5-defined PTSD symptoms due to the COVID-19 pandemic. The PCL-5 is a 20-item self-report instrument that was rated on a 5-point Likert scale (*0* = *not at all;4* = *extremely*). Responses were summed (range: 0–80), with higher scores indicating greater PTSD symptom severity. Participants with scores >33 were considered to have clinically relevant PTSD symptoms. The Chinese version of PCL-5 has been validated among Chinese populations including adolescents (38). Internal consistency was α = 0.973 for the PCL-5 in this sample.

### Statistical analysis

Descriptive statistics summarized the sociodemographic characteristics and COVID-19 and mental disorder-related stigma responses using means and standard deviation. To compare stigmatizing views about the two health conditions by sociodemographic variables, we conducted independent-sample *t*-tests and analyses of variance (ANOVA). We also estimated the means and standard deviation for each stigma scale item. In the Public Stigma of COVID-19 Scale, individuals who responded with *agree* or *strongly agree* were considered to endorse stigmatizing views. In the LPDDS, endorsement was defined as answering *disagree* or *strongly disagree*, except for reverse-scored items, where answering *agree* or *strongly agree* was considered an endorsement. A score of 2.5 was used as a midpoint cutoff for the two stigma scales and mean responses of >2.5 were considered an endorsement of stigmatizing views. In terms of the total stigma score, respondents who reported average scores >2.5 were treated as endorsing public stigma. We present the prevalence of endorsement for each item and for each total stigma scale. Association between the two stigma scores and mental health-related factors (GHQ-12, PHQ-9, GAD-7, ISI-7, and PCL-5) were tested with independent-sample *t*-tests. The variables significantly associated with either stigma scale were included in multiple linear regressions as dependent variables to identify factors influencing stigmatizing views. Data were analyzed using SPSS v.26.

## Results

### Sociodemographic characteristics and bivariate relationships with stigma scales

A total of 9,759 students participated in the survey and 9,749 adolescent students (99.9%) who had finished all relevant scale questionnaires were included in this study. Table 1 shows their sociodemographic characteristics and the mean scores and standard deviations of the COVID-19-related stigma and mental disorder-related stigma scales by sociodemographic variables. A majority of the participants were female students (59.4%), were aged between 18 and 24 years (73.5%), had siblings (68.3%), and reported an average-level family income (76.0%). Nearly 9 in 10 (87.9%) students were not quarantined during the COVID-19 pandemic and only 14.8% reported a history of mental disorders.

**Table 1:** Sociodemographic characteristics and stigma scores of participants.

		**COVID-19-related stigma score**			**Mental disorder-related stigma score**		
**Characteristic**	***N*** **(%)**	**Mean**	**SD**	***P***	**Mean**	**SD**	***P***
Total	9,749 (100)	21.90	6.31		31.02	5.16	
**Gender**				<0.001^***^			0.539
Male	3,954 (40.6)	22.29	6.68		31.15	5.23	
Female	5,795 (59.4)	21.64	6.03		30.93	5.10	
**Age group**							
15–17	2,579 (26.5)	22.29	6.44	0.009^**^	30.78	5.59	<0.001^***^
18–24	7,170 (73.5)	21.76	6.25		31.11	4.99	
**Education attainment**				<0.001^***^			0.014^*^
Junior high school	130 (1.3)	22.27	7.01		29.84	6.24	
Senior high school	2,378 (24.4)	22.46	6.42		30.86	5.55	
College	4,880 (50.1)	22.52	6.57		31.09	4.79	
Undergraduate	2,361 (24.2)	20.05	5.14		31.11	5.39	
**Family income level**				0.001^**^			<0.001^***^
<average level	1,856 (19.0)	22.23	6.37		31.30	5.09	
= average level	7,407 (76.0)	21.87	6.27		31.02	5.12	
>average level	486 (5.0)	21.09	6.55		30.02	5.77	
**One-child or not**				0.363			<0.001^***^
Yes	3,087 (31.7)	21.89	6.34		30.98	5.62	
No	6,662 (68.3)	21.91	6.29		31.04	4.93	
**Quarantine condition**				0.012^*^			0.105
Quarantine	1,181 (12.1)	21.13	5.95		31.19	5.26	
Without quarantine	8,568 (87.9)	22.00	6.35		31.00	5.14	
**History of mental disorders**				0.075			<0.001^***^
No or unknown	8,303 (85.2)	21.85	6.26		30.91	5.01	
Yes	1,446 (14.8)	22.21	6.57		31.68	5.86	

^*^p <0.05, ^**^p <0.01, ^***^p <0.001.

SD, standard deviation.

Significant differences were found in bivariate analyses. Higher COVID-19-related stigma scores were significantly associated with male sex (*t* = −5.002, *p* < 0.001), age ranging from 15 to 17 years (*t* = 3.631, *p* < 0.01), college-level educational attainment (*F* = 92.555, *p* < 0.001), below-average family income (*F* = 6.633, *p* < 0.01), and never being quarantined (*t* = −4.508, *p* < 0.05). Mental disorder-related stigma scores were significantly associated with age ranging from 18 to 24 years (*t* = −2.778, *p* < 0.001), undergraduate-level educational attainment (*F* = 3.545, *p* < 0.05), below-average family income (*F* = 11.789, *p* < 0.001), not being a single child (*t* = −0.533, *p* < 0.001), and a history of mental disorders (*t* = −5.266, *p* < 0.001).

### Endorsement of individual stigma scale items

Table 2 presents the means, standard deviations, and prevalence of endorsement for each item of the two stigma scales and for the full scales. Only two COVID-19-related stigma scale items were endorsed by over half of the sample, including “I will intentionally try my best to keep a distance from people with COVID-19” (91.2%) and “I am worried that people with COVID-19 will cause harm to others” (78.5%). These items also had the highest mean scores (3.51 and 3.02, respectively). The total mean score for the COVID-19-related stigma scale was 1.99, with 17.2% of participants endorsing COVID-19-related stigma.

**Table 2:** Endorsement of stigma items related to COVID-19 and mental disorders.

	**Mean (SD)**	**Percent endorsing (%)**
COVID-19-related stigma	1.99 (0.57)	17.2
I am worried that people with COVID-19 will cause harm to others.	3.02 (0.88)	78.5
I will intentionally try my best to keep a distance from people with COVID-19.	3.51 (0.77)	91.2
People with COVID-19 are revolting.	1.63 (0.82)	14.5
People with COVID-19 are a burden to society.	1.50 (0.95)	11.4
People with COVID-19 will cause trouble to others.	1.98 (0.96)	30.3
People with COVID-19 should stay away from others, even if he/she has been cured.	1.67 (0.85)	15.8
It is normal for people with COVID-19 to be discriminated against by others.	1.42 (0.71)	9.1
People who have been infected with covid-19 are not suitable for cooking, education, healthcare and childcare-related work.	1.70 (0.87)	18.6
When I interact with people with COVID-19, I am worried that I will be infected with COVID-19, even if he/she has been cured.	1.95 (0.90)	28.8
I would avoid socializing with someone infected with COVID-19, even if he/she has been cured.	1.80 (0.86)	20.7
I refuse to have physical contact with someone infected with COVID-19, even if he/she has been cured.	1.73 (0.84)	17.8
Mental disorder-related stigma	2.39 (0.40)	40.7
Most people would accept a person with psychosis as a close friend.	2.41 (0.80)	47.5
Most people believe that a person with psychosis is just as intelligent as the average person.	2.33 (0.79)	41.1
Most people believe that a person with psychosis is just as trustworthy as the average citizen.	2.35 (0.79)	43.8
Most people would accept a person with psychosis as a teacher of young children in a public school.	2.39 (0.83)	46.1
Most people believe that entering a psychiatric hospital is a sign of personal failure (R).	2.00 (0.83)	79.5
Most people will not hire a person with psychosis to take care of their children even if he or she had been well for some time (R).	2.55 (0.83)	45.2
Most people think less of a person with psychosis (R).	2.22 (0.84)	67.1
Most employers will hire a person with psychosis if he or she is qualified for the job	2.54 (0.80)	54.4
Most employers will pass over the application of a person with psychosis in favor of another applicant (R).	2.51 (0.80)	47.5
Most people in my community would treat a person with psychosis just as they would treat anyone.	2.33 (0.87)	39.3
Most young people would be reluctant to date someone with psychosis (R).	2.44 (0.78)	54.1
Once they know a person is a patient with psychosis, most people will take his or her opinions less seriously (R).	2.39 (0.79)	57.2
Most people think that a person with psychosis is dangerous and unpredictable (R).	2.57 (0.80)	43.5

SD, standard deviation. Items marked with (R) were reversed.

Regarding mental disorder-related stigma, over half of the participants endorsed five LPDDS items, stating that most people believe that entering a psychiatric hospital is a sign of personal failure (79.5%), think less of a person with psychosis (67.1%), will take a person's opinions less seriously once they know she/he is a patient with psychosis (57.2%), and would be reluctant to date with people with mental disorders (54.1%). For the full scale, the total mean score was 2.39, with 40.7% of the participants endorsing stigmatizing views of mental disorders.

### Bivariate associations between mental health-related variables and stigma

Associations between mental health-related variables and the stigma scales are presented in Table 3. Individuals with health problems (*r* = −3.469, *p* < 0.05) and PTSD symptoms (*r* = −5.954, *p* < 0.001) had higher COVID-19-related stigma scores. By contrast, respondents with health problems (*r* = −8.502, *p* < 0.05) and symptoms of depression (*r* = −11.639, *p* < 0.001), anxiety (*r* = −11.348, *p* < 0.001), and insomnia (*r* = −8.277, *p* < 0.05) reported higher mental disorder-related stigma scores.

**Table 3:** Correlation of mental health-related factors and scores of two public stigma scales.

**Mental health-related factors**	**COVID-19-related stigma score**		**Mental disorder-related stigma score**	
	**Mean (SD)**	***P***	**Mean (SD)**	***P***
**GHQ-12, mental health problems**		0.043^*^		0.047^*^
No	21.78 (6.37)		30.79 (5.06)	
Yes	22.32 (6.08)		31.85 (5.40)	
**PHQ-9, depression symptoms**		0.146		<0.001^***^
No	21.63 (6.35)		30.60 (5.17)	
Yes	22.47 (6.18)		31.89 (5.02)	
**GAD-7, anxiety symptoms**		0.383		<0.001^***^
No	21.61 (6.32)		30.66 (5.17)	
Yes	22.70 (6.20)		31.99 (4.99)	
**ISI, insomnia symptoms**		0.075		0.026^*^
No	21.68 (6.37)		30.76 (5.14)	
Yes	22.48 (6.12)		31.71 (5.14)	
**PCL-5, PTSD symptoms**		<0.001^***^		0.301
No	21.82 (6.24)		30.94 (5.11)	
Yes	23.70 (7.54)		32.89 (5.81)	

^*^p <0.05, ^***^p <0.001.

SD, standard deviation; GHQ, General Health Questionnaire; PHQ, Patient Health Questionnaire; GAD, General Anxiety Disorder; ISI, Insomnia Severity Index; PCL, PTSD (Post-traumatic Stress Disorder) Checklist.

### Factors associated with stigma related to COVID-19 and mental disorders

Multiple linear regression analyses (Table 4) showed that male sex (β = 0.025, *p* < 0.05), a lack of quarantine experience (β = 0.035, *p* < 0.001), below undergraduate level education attainment (β = 0.046–0.188, *p* < 0.001), and average (β = 0.066, *p* < 0.01) and below-average family income (β = 0.052, *p* < 0.01) were associated with higher COVID-19-related stigma scores. On the LDDPS, average (β = 0.065, *p* < 0.01) or below-average (β = 0.065, *p* < 0.01) family income, general mental health problems (β = 0.027, *p* < 0.05), and symptoms of depression (β = 0.056, *p* < 0.001) and anxiety (β = 0.051, *p* < 0.001) were associated with higher mental disorder-related stigma scores.

**Table 4:** Factors associated with public stigma toward COVID-19 and mental disorders.

	**Variables**	***B***	**SE B**	**β**
**COVID-19-related stigma**	Gender (0 = female)			
	Male	0.315	0.131	0.025^*^
	Age group (0 = 15–17)			
	18–24	0.464	0.282	0.032
	Education attainment (0 = undergraduate)			
	Junior high school	2.536	0.619	0.046^***^
	Senior high school	2.768	0.305	0.188^***^
	College	2.287	0.159	0.181^***^
	Family income level (0 = >average level)			
	<average level	1.054	0.322	0.066^**^
	= average level	0.770	0.292	0.052^**^
	Quarantine condition (0 = Quarantine)			
	Without Quarantine	0.675	0.193	0.035^***^
	GHQ-12, healthy problem (0 = No)			
	Yes	0.271	0.157	0.018
	PCL-5, PTSD symptoms (0 = No)			
	Yes	1.402	0.322	0.045^***^
**Mental disorder-related stigma**	Age group (0 = 15–17)			
	18–24	0.342	0.232	0.029
	Education attainment (0 = undergraduate)			
	Junior high school	−0.852	0.509	−0.019
	Senior high school	−0.001	0.251	0.000
	College	−0.040	0.129	−0.004
	Family income level (0 = >average level)			
	<average level	0.849	0.267	0.065^**^
	= average level	0.783	0.242	0.065^**^
	One-child or not (0 = Yes)			
	No	−0.076	0.115	−0.007
	History of mental disorders (0 = No or unknown)			
	Yes	−0.217	0.154	−0.015
	GHQ-12, mental health problems (0 = No)			
	Yes	0.337	0.147	0.027^*^
	PHQ-9, depression symptoms (0 = No)			
	Yes	0.617	0.165	0.056^***^
	GAD-7, anxiety symptoms (0 = No)			
	Yes	0.596	0.167	0.051^***^
	ISI, insomnia symptoms (0 = No)			
	Yes	0.157	0.140	0.014

^*^p <0.05, ^**^p <0.01, ^***^p <0.001.

B, Unstandardized Coefficients; SE B, Standard Error of Unstandardized Coefficients; β, Standardized Coefficients; GHQ, General Health Questionnaire; PHQ, Patient Health Questionnaire; GAD, General Anxiety Disorder; ISI, Insomnia Severity Index; PCL, PTSD (Posttraumatic Stress Disorder) Checklist.

## Discussion

Extending from previous studies about health-related stigma only focusing on one health condition (39), this is the first study that simultaneously focused on stigma related to both COVID-19 and mental disorders. The current study compared the magnitude of stigmatizing attitudes toward COVID-19 and mental disorders as well as explored factors associated with two types of stigmas. The findings of this study indicate that people with COVID-19 or mental disorders are stigmatized by young people to varying degrees, with the stigma attached to mental disorders being more prevalent. The study findings also contribute to understanding the association between influencing factors on COVID-19- and mental disorder-related stigmas.

We found that the stigma of mental disorders was higher in adolescents and young adult students than the stigma of COVID-19, with 17.2% of the participants endorsing stigmatizing views toward people with COVID-19, whereas 40.7% of the participants had a mental disorder-related stigma. Looking at the specific items that the participants responded to, we found that 50–80% of the participants had stereotypes toward people with mental disorders, such as being diagnosed with mental disorders is a sign of personal failure (79.5%). Apart from adverse cognition, more than half of the participants tended to refuse people with mental disorders in social life, such as reluctance to date people with mental disorders (54.1%). In terms of COVID-19, only two items scored higher than 50%. Given that the age of participants in this study was 15–24 years, this was not a surprising result because previous studies have found that those who were younger were less likely to have stigmatizing attitudes toward COVID-19 (40). In addition, considering the strict prevention and control policies for COVID-19 in China, the number of COVID-19 infected cases was only two during the study period and the COVID-19 did not pose a threat to participants. Therefore, lower levels of stigma related to COVID-19 may be linked to decreased levels of threat.

Previous studies have also shown that discrepancies in the stigma level in different illnesses were related to features of diseases (24). A previous study among adolescents showed that mental disorder was stigmatized more than other health conditions (41). Unlike COVID-19, which is contagious but can be cured with medication, a mental disorder is a chronic condition with common misconceptions (e.g., dangerous, irresponsible, and childlike) perceived by the general population. Moreover, the lack of mental disorders knowledge among adolescents might be associated with stigmatization attitudes toward people with mental disorders. Given the cultural values in China, such as Confucianism, face concern, and etiological beliefs about mental disorders, the stigmatizing attitudes would be intensified (2, 42). Future studies are needed to explore what might account for the different magnitudes among various health conditions.

Among sociodemographic variables, the current study indicated that girls and young women had a lower level of stigma toward people with COVID-19, which is consistent with the finding from a previous study that boys and young men endorsed more COVID-19 stigma than their female counterparts (10). In terms of mental disorder-related stigma, however, this gender discrepancy was not found. Based on previous literature about the role of gender on stigma, several studies have indicated that female students hold lower levels of stigma toward individuals with certain mental disorders (43), while others have found that female students were also inclined to endorse more unfavorable attitudes (44). Given the lack of consistent findings on the association between gender and stigma toward people with mental disorders, this research area merits further investigation.

The results of this study showed that young adults over 18 years were less likely to endorse COVID-19-related stigma than adolescents aged 15–17 years. This finding was contrary to previous studies among the adult population indicating that those who were older had great odds of holding a stigma attached to SARS or COVID-19 (29, 40). While these inconsistent results may not be surprising, given that the lower age group of participants in the current study was relatively young without psychological maturity resulting in shaping more negative attitudes toward people with COVID-19 (13). In addition, youths over 18 years with a higher level of education could be more capable of acquiring knowledge about COVID-19 and thus contribute to less stigma (45). This coincided with our finding that participants with an undergraduate degree reported lower levels of COVID-19-related stigma compared with those having a lower education level. With regard to mental disorder-related stigma, interestingly, participants over 18 years and with an undergraduate degree were more likely to hold stigmatizing attitudes. This may be related to the fact that college students are more likely to be influenced by social culture, thereby shaping the adverse cognition toward people with mental disorders during socialization. Other evidence indicated that mental health literacy was low among Chinese college students, which might be associated with the high level of stigma attached to mental disorders as well (46).

One promising finding was that participants with above-average family income were less likely to endorse stigmatizing attitudes toward people with COVID-19. Evidence from a previous study has indicated that children with better family economic status may receive sufficient instrumental and emotional support to cope with COVID-19 and thus face less psychological distress (47). Another research has demonstrated that better mental health status was associated with less COVID-19-related discrimination and stigma (48). Hence, a higher family income may decrease the likelihood of endorsing COVID-19-related stigma. However, contrary to previous research among the general population, participants with a poorer family income level in this study reported higher levels of mental disorder-related stigma. This result is in line with the finding from a study among college students which found that perceived public stigma was higher among individuals with lower socioeconomic status (49). These findings of stigma discrepancy by youth with different levels of family income may be helpful for identifying a target intervention group to diminish stigma.

This study also found that participants without quarantine experience were more likely to endorse COVID-19-related stigma. Thus, people experiencing a quarantine situation might build more empathy for those with COVID-19; as a result, those with quarantine experience had lower odds of endorsing stigma. In addition, this study found greater mental disorder-related stigma among participants with a positive history of mental disorders. Previous studies have shown that people with mental disorders might internalize negative public stereotypical views and feel inferior to others (48), and other evidence indicated that people with mental disorders would encounter overt discrimination in society (50). Consequently, people with a history of mental disorders were more prone to have stigmatized views given the dual effects of social discrimination and internalization of negative notions.

In terms of the association between mental health conditions and stigma, the results of our study showed that participants with PTSD symptoms were more likely to endorse COVID-19-related stigma. This is consistent with previous studies pertaining to COVID-19-related stigma and PTSD, which found that higher levels of stigma toward people with COVID-19 were significantly positively correlated with severe symptoms of PTSD (51). However, this significant association was not found regarding mental disorder-related stigma. Our study found that stigmatization related to mental disorders was significantly correlated to depression and anxiety symptoms. This finding may give credence to the notion that the stigma of mental disorders may be intertwined with COVID-19 (52). Previous studies have shown that young people suffered from depression, anxiety, and other psychological distress during the COVID-19 pandemic, which might contribute to specific discrimination (48). Whether the epidemic of COVID-19 would increase the prevalence of mental disorder-related stigma remains unknown; therefore, more future empirical validation will be needed.

There are limitations to this study that should be acknowledged. First, our convenient sampling strategy, while sampling youth in one city in central China, will limit the generalizability of the results. Second, given the cross-sectional design, our study cannot test the causality. To elucidate the predictor of stigma attached to mental disorders and COVID-19, a longitudinal design should be considered in the future. Finally, several other factors which were not included in this study may be correlated with stigmatizing attitudes, such as knowledge and attribution of the disease (28). Future research should examine these factors to make a comprehensive understating of the stigma mechanism attached to various health conditions.

## Conclusion

Despite these limitations, the results of this study furnish a description for understanding the distinction of the stigma profile between two health conditions (COVID-19 and mental disorders). Findings showed that the stigma of mental disorders is higher in this youth population than the stigma of COVID-19. Our findings shed light on the need for tailoring a targeted intervention for stigma reduction (53). Groups that should be prioritized for ameliorating stigmatizing attitudes toward COVID-19 include boys and young men, youth with lower educational levels, and those with PTSD symptoms. With respect to mental disorder-related stigma, the target intervention group should focus on youths with an undergraduate degree and those with depression and anxiety symptoms. Given the deleterious impact of the prolonged COVID-19 pandemic on mental health among young people, it might increase the stigma and discrimination against both COVID-19 and mental disorders. Thus, policymakers and researchers should design comprehensive programs to ameliorate psychological distress and combat stigma among youth.

## Data availability statement

The raw data supporting the conclusions of this article will be made available by the authors, without undue reservation.

## Ethics statement

The studies involving human participants were reviewed and approved by Medical Ethics Committee of the Jingzhou Mental Health Center. Written informed consent to participate in this study was provided by the participants' legal guardian/next of kin.

## Author contributions

M-SR and BL designed this study. BL, M-SR, X-PD, and X-FZ conducted this study. BL, X-PD, X-FZ, Y-YF, C-CL, and LH collected data. T-MZ conducted data analysis. T-MZ and M-SR wrote the first draft of the manuscript. All authors made contributions to the critical revision of the manuscript. All authors contributed to the article and approved the submitted version.
